# Supplementary material for: RapidRat: Development, validation and application of a genotyping-by-sequencing panel for rapid biosecurity and invasive species management
Source: PLoS One. 2020 Jun 18;15(6):e0234694. doi: 10.1371/journal.pone.0234694 (PMC7302687; doi:10.1371/journal.pone.0234694)
Supplement: Attachment — Submitted filename: Sjodin_et_al_response_PLOSONE.pdf [file pone.0234694.s004.pdf]

Reviewer #1
Comments to the Author:
The paper presents novelty and describes the way the authors obtained genomic data to evalute invasive species. The authors developed, validated and deployed an effective and efficient genotyping tool (RapidRat) for informing invasive rat management in Haida Gwaii.

To improve the paper I recomend:
a) Make the results clear in the abstract section

RESPONSE: We have added details regarding the number of source SNPs from ddRAD and the assignment probability of the individuals of unknown origin (lines 22,27-28).

b) Introduction and material and methods: are Ok.

c) Results: ok

d) Discussion and conclusions: Improve them to clarify the novelty, importance and the limitations of such research

RESPONSE: We have added two paragraphs to the Discussion (lines 323-342) highlighting: 1) limitations (namely, the requirement for existing genetic data for SNP identification and primer design); and novelty/importance. Regarding the latter, we summarized some applications of GT-seq to inform management and conservation, while noting that ours is the first study to apply GT-seq for informing invasive species management.

Reviewer #2
Comments to the Author:
The manuscript entitled "RapidRat: development, validation and application of a genotyping-by-sequencing panel for rapid biosecurity and invasive species management" describes the development of a rat specific panel for genomic data to access information about invasive species of rats in an archipelago in British Columbia, Canada. The authors proposed a novel and cheaper way to genotype individuals of brown and black rats using the detection of SNPs polymorphisms to track novel and old rat invasions in the islands.

I recommend this manuscript to be accepted for publication in PlosOne after the review of the following suggestions:

I am really concerned about all figures, graphs and map. I suggest to improve the quality of the images since all of them show bad aspect, with lots of pixels. I also, recommend to improve the map with clear information of the specific islands used to test the novel panel.

RESPONSE: All Figures have been re-generated to a higher quality. We have also indicated on the map which islands (Agglomerate, Ramsay, and Hotspring; written in red) were used for panel validation.

Introduction:
In general, I suggest to add some information about the movement of the rats between islands and continent to island. How do the rats move to island to island? Is it only by stowaways' ships or is there another hypothesis? I missed some information about the distance between the island (especially the ones that the authors used to test the novel panel with the recent rat invasions). Also, I am concerned about the movement of tourists, people and ships between island. Do the people still practice fishing? Do they moor the boat in the islands? Are there big or small boats?

RESPONSE: Haida Gwaii is located ~80 km from the mainland (added to line 98), so the most likely vector of dispersal to the islands from the mainland is as stowaways (indicated on lines 101-103). As for inter-island dispersal, rats can and likely do swim in these temperate waters,

potentially up to 1 km (lines 350-353). Other methods of dispersal include rafting on debris during high tide and as stowaways on ships. There is significant fishing and logging throughout the islands using boats of various size which could serve as vectors for rat dispersal. This information has been added to the methods section as well as distance between islands for novel invasions (lines 113-119).

Line 47: The term "disproportionately" should be better explained. Do "high levels of biodiversity" change between island or between island and continent?

RESPONSE: These differences in biodiversity are between island and mainland; this point has been better clarified in the revised manuscript (lines 47-49).

Line 75-79: This sentence needs to be improved for a better explanation of how they performed the hypothesis on Pearl Island.

RESPONSE: The authors used multiple analyses of population structure as well as population assignment tests to assess the genetic origin of the post-eradication population on Pearl Island. We have edited this sentence for clarity as suggested (line 76).

Methods:
Line 100: Are the island breeding habitat for other animals as well?

RESPONSE: The islands are home to many species; however, rats are having the most severe impact on ground-nesting seabirds, hence the focus of seabird breeding in this manuscript.

Line 133: I suggest to change the sentence "See Table 1 for detailed sample distribution." to the previous paragraph where you explain the geographic distribution and the number of used samples.

RESPONSE: This sentence has been moved the previous section (line 133-134) for improved clarity.

Line 163: Do you incorporate all islands that the rats were found? How about the bigger islands? Was all the geographic distribution contemplate? I mean, are there more than one population of rats in the bigger island?

RESPONSE: Our sample does not include all islands where rats are present, though they do approximate the overall distribution of rats across the islands (lines 166-168). As to the second half of the question, multiple sites were sampled on the larger islands; however, unless otherwise noted, all sites on a given island were highly connected and acted as a single genetic population based on our previous work (Sjodin *et al*. 2019 *Evolutionary Applications*), which is noted here (lines 122-123).

Line 172: What was the specific Miseq sequence reagent used?

RESPONSE: We did not perform sequencing in-house, so do not know the precise reagents used for the MiSeq run. In response, we have added the name of the facility that sequenced the library (line 175-177).

Line 200: The word "iterations" are misspelling.

RESPONSE: This is the correct spelling as per the Merriam-Webster and Cambridge dictionaries.

Results:
Line 221: I understand that you use 315 and 429 loci for brown and black respectively from the 708 putative loci. From the 708 loci, 8 of them were species-specific. Do the 8 species-specific loci remain in the 315 and 429 loci used? If so, I think it needs to be better described. Also, it needs to be better explain if the species-specific mentioned is a combined loci. In the same way, I wonder if there is another way to confirm the black and brown rat species, like barcode DNA?

RESPONSE: Thank you for highlighting these points. We have added more details to the first paragraph of the results to provide further clarity, including that three of the eight loci remained after optimization in our final 443 loci panel, retained in both the brown and black rat specific panels (lines 226-227). Additionally, we now explicitly report: 1) 100% species assignment concordance with the ddRAD results; 2) significant differentiation between brown and black rats for all 443 loci; 3) 150 of 443 loci had a $\theta > 0.90$ (lines 226-232).

Item 241:
How about the loss of finer-scale structure in Kunghit?

RESPONSE: We did not find fine-scale structure for black rats on Kunghit Island using the full ddRAD dataset, so there was no loss of resolution using the GT-seq panel (fine-scale structure was only seen for brown rats on Kunghit Island).

Discussion:
Line 269-278: I suggest to improve the discussion about the hypotheses of how the rats invade the island as I suggested in the introduction. In this paragraph, I understood that the rats have the capacity of swim in the temperate waters. Is that possible?

RESPONSE: Brown rats can swim in temperate waters, though the exact maximum distance is unknown (potentially >1km according to some estimates). They also can disperse via "rafting", or floating on debris from island to island, especially during high tide. And as always, commensal dispersal via boats is a possibility. This information has been added to lines 285-288.

Line 303-304: I think is important to point out the need to perform ddRAD-seq previously in the species that the researches are interested to apply GT-seq. In this manuscript, there was already been developed the ddRAD-seq to analyze the genetic population of those rats, but in most case, this is a very consuming stage.

RESPONSE: We have added a section to the Discussion (lines 323-333) highlighting these points.
