# Supplementary material for: RapidRat: Development, validation and application of a genotyping-by-sequencing panel for rapid biosecurity and invasive species management
Source: PLoS One. 2020 Jun 18;15(6):e0234694. doi: 10.1371/journal.pone.0234694 (PMC7302687; doi:10.1371/journal.pone.0234694)
Supplement: Attachment — Submitted filename: Review.docx [file pone.0234694.s003.docx]

The manuscript entitled “RapidRat: development, validation and application of a genotyping-by-sequencing panel for rapid biosecurity and invasive species management” describes the development of a rat specific panel for genomic data to access information about invasive species of rats in an archipelago in British Columbia, Canada. The authors proposed a novel and cheaper way to genotype individuals of brown and black rats using the detection of SNPs polymorphisms to track novel and old rat invasions in the islands.

I recommend this manuscript to be accepted for publication in PlosOne after the review of the following suggestions:

I am really concerned about all figures, graphs and map. I suggest to improve the quality of the images since all of them show bad aspect, with lots of pixels. I also, recommend to improve the map with clear information of the specific islands used to test the novel panel.

Introduction:

In general, I suggest to add some information about the movement of the rats between islands and continent to island. How do the rats move to island to island? Is it only by stowaways’ ships or is there another hypothesis? I missed some information about the distance between the island (especially the ones that the authors used to test the novel panel with the recent rat invasions). Also, I am concerned about the movement of tourists, people and ships between island. Do the people still practice fishing? Do they moor the boat in the islands? Are there big or small boats?

Line 47: The term “disproportionately” should be better explained. Do “high levels of biodiversity” change between island or between island and continent?

Line 75-79: This sentence needs to be improved for a better explanation of how they performed the hypothesis on Pearl Island.

Methods:

Line 100: Are the island breeding habitat for other animals as well?

Line 133: I suggest to change the sentence “See Table 1 for detailed sample distribution.” to the previous paragraph where you explain the geographic distribution and the number of used samples.

Line 163: Do you incorporate all islands that the rats were found? How about the bigger islands? Was all the geographic distribution contemplate? I mean, are there more than one population of rats in the bigger island?

Line 172: What was the specific Miseq sequence reagent used?

Line 200: The word “iterations” are misspelling.

Results:

Line 221: I understand that you use 315 and 429 loci for brown and black respectively from the 708 putative loci. From the 708 loci, 8 of them were species-specific. Do the 8 species-specific loci remain in the 315 and 429 loci used? If so, I think it needs to be better described. Also, it needs to be better explain if the species-specific mentioned is a combined loci. In the same way, I wonder if there is another way to confirm the black and brown rat species, like barcode DNA?

Item 241:

How about the loss of finer-scale structure in Kunghit?

Discussion:

Line 269-278: I suggest to improve the discussion about the hypotheses of how the rats invade the island as I suggested in the introduction. In this paragraph, I understood that the rats have the capacity of swim in the temperate waters. Is that possible?

Line 303-304: I think is important to point out the need to perform ddRAD-seq previously in the species that the researches are interested to apply GT-seq. In this manuscript, there was already been developed the ddRAD-seq to analyze the genetic population of those rats, but in most case, this is a very consuming stage.
